# Endocrine Dysfunction in Children with Zika-Related Microcephaly Who Were Born during the 2015 Epidemic in the State of Pernambuco, Brazil

**DOI:** 10.3390/v13010001

**Published:** 2020-12-22

**Authors:** Andréia Veras Gonçalves, Demócrito de B. Miranda-Filho, Líbia Cristina Rocha Vilela, Regina Coeli Ferreira Ramos, Thalia V. B. de Araújo, Rômulo A. L. de Vasconcelos, Maria Angela Wanderley Rocha, Sophie Helena Eickmann, Marli Tenório Cordeiro, Liana O. Ventura, Ulisses Ramos Montarroyos, Alessandra Mertens Brainer, Maria Durce Costa Gomes, Paula Fabiana Sobral da Silva, Celina M. T. Martelli, Elizabeth B. Brickley, Ricardo A. A. Ximenes

**Affiliations:** 1 Department of Tropical Medicine, Health Sciences Center, Federal University of Pernambuco, Recife 50670-901, Brazil; mouralibia@gmail.com (L.C.R.V.); raaximenes@uol.com.br (R.A.A.X.); 2 Unit Endocrinology, Oswaldo Cruz University Hospital, Recife 50100-130, Brazil; 3 Postgraduation in Health Sciences, University of Pernambuco, Recife 50100-010, Brazil; demofilho@gmail.com (D.d.B.M.-F.); romulo.alv@hotmail.com (R.A.L.d.V.); ulisses.montarroyos@upe.br (U.R.M.); mertensbrainer@yahoo.com.br (A.M.B.); mdurce2@gmail.com (M.D.C.G.); paulafsobral@yahoo.com.br (P.F.S.d.S.); 4 Department Pediatric Infectious Disease, Oswaldo Cruz University Hospital, Recife 50100-130, Brazil; coeliramos@hotmail.com (R.C.F.R.); mangelarocha@uol.com.br (M.A.W.R.); 5 Department of Social Medicine, Federal University of Pernambuco, Recife 50670-901, Brazil; thalia.velhobarreto@gmail.com; 6 Maternal and Child Department, Health Sciences Center, Federal University of Pernambuco, Recife 50670-901, Brazil; sophie.eickmann@gmail.com; 7 Unit of Oswaldo Cruz Foundation (Fiocruz), Research Center Aggeu Magalhães (CPqAM), Recife 50670-420, Brazil; marli.tenorio@gmail.com (M.T.C.); turchicm@gmail.com (C.M.T.M.); 8 Department of Ophthalmology, Altino Ventura Foundation, Recife 52171-011, Brazil; lianaventuramd@gmail.com; 9 Department of Infectious Disease Epidemiology, London School of Hygiene & Tropical Medicine, London WC1E 7HT, UK; Elizabeth.Brickley@lshtm.ac.uk; 10 Department of Internal Medicine, University of Pernambuco, Recife 50100-010, Brazil

**Keywords:** microcephaly, Zika, obesity, hypothyroidism, puberty

## Abstract

Congenital viral infections and the occurrence of septo-optic dysplasia, which is a combination of optic nerve hypoplasia, abnormal formation of structures along the midline of the brain, and pituitary hypofunction, support the biological plausibility of endocrine dysfunction in Zika-related microcephaly. In this case series we ascertained the presence and describe endocrine dysfunction in 30 children with severe Zika-related microcephaly from the MERG Pediatric Cohort, referred for endocrinological evaluation between February and August 2019. Of the 30 children, 97% had severe microcephaly. The average age at the endocrinological consultation was 41 months and 53% were female. The most frequently observed endocrine dysfunctions comprised short stature, hypothyroidism, obesity and variants early puberty. These dysfunctions occurred alone 57% or in combination 43%. We found optic nerve hypoplasia (6/21) and corpus callosum hypoplasia (20/21). Seizure crises were reported in 86% of the children. The most common—and clinically important—endocrine dysfunctions were pubertal dysfunctions, thyroid disease, growth impairment, and obesity. These dysfunctions require careful monitoring and signal the need for endocrinological evaluation in children with Zika-related microcephaly, in order to make early diagnoses and implement appropriate treatment when necessary.

## 1. Introduction

Congenital Zika virus (ZIKV) infections have been primarily characterized in terms of their impact on central nervous system development [1,2,3,4]. Among children with Zika-related microcephaly, midline defects, such as corpus callosum dysgenesis and pellucid septum [2,5], and significant ophthalmic abnormalities with optic nerve involvement, including optic nerve hypoplasia and optic nerve pallor [6] have been described. As these alterations, together referred to as septo-optic dysplasia, are known to form a classic triad with hypothalamus–pituitary axis dysfunction [7,8,9,10], we hypothesized that endocrine dysfunction may present in children with congenital Zika syndrome (CZS).

Although the clinical presentation has not yet been fully established and much research is still underway with the aim of fully characterizing this new syndrome, studies from other congenital viral infections support the biological plausibility of endocrine dysfunction in CZS. Specifically, children with congenital rubella infections have been reported to have higher risk of diabetes mellitus, thyroid dysfunction, growth hormone deficiency, and Addison’s disease [11,12]. Similarly, children with congenital cytomegalovirus infections, which may cause severe brain damage resembling that of CZS [3], may develop endocrine disorders [13,14]. A comprehensive neuropathological study in ten newborn babies infected with ZIKV during pregnancy showed focal microscopic calcification in two pituitaries examined [15]. 

Wu et al. showed that ZIKV can infect the hypothalamus, causing multi-hormonal deficiencies and delayed growth and development in a mouse model [16]. A study using a porcine conceptuses model that partially reproduces persistent ZIKV infection in utero, tested the levels of cortisol in the amniotic fluid from inoculated conceptuses at the beginning and in the middle of pregnancy. This study demonstrated that persistent ZIKV infection in pigs is associated with high levels of cortisol in amniotic fluids [17].

Therefore, to ascertain whether endocrine and/or hypothalamic–pituitary axis dysfunctions are present in CZS, the present study clinically evaluated children with Zika-related microcephaly born during the 2015 epidemic in the state of Pernambuco, Brazil. The children in this cases series represent participants in the Microcephaly Epidemic Research Group Pediatric Cohort (MERG-PC) who were referred for suspected endocrinological dysfunction. 

## 2. Materials and Methods 

The study involving children with Zika-related microcephaly was reviewed and approved by the Research Ethics Committee at the Hospital Universitário Oswaldo Cruz approval on 10 October 2016 (CAAE52803316800005192), amended on 30 August 2019. Written informed consent to participate in this study was provided by the participants’ legal guardian/next of kin. 

Between February and June 2019, children with Zika-related microcephaly participating in the MERG-PC were referred for evaluation of suspected endocrinological dysfunction to the pediatric infectious disease section of the Pernambuco State University Hospital. Patients’ parents or guardians were informed of this investigation during outpatient consultations, and those who agreed to their children’s participation signed an informed consent form. 

The clinical team completed the study questionnaire with information from anamnesis and physical examination. Premature births were defined as those occurring at <37 weeks of gestational age. Children were considered to be small for gestational age (SGA) with a birthweight below the tenth percentile by gestational age and low birthweight with a birthweight of <2500 g. Z-scores for head circumference, length, weight, and BMI were calculated using the World Health Organization (WHO) Anthropometric calculator, Anthro software version 3.2.2 tool [18]. Head circumference was measured using an inelastic tape measure positioned over the occipital prominence and the eyebrow arch. With the child’s head fixed, the tape was placed firmly around the frontal bone over the supraorbital groove and then wrapped around the head, at the same level on each side, over the maximal occipital prominence. Microcephaly cases were considered to be severe when the head circumference z-score was ≤−3 standard deviations of the growth curves established according to sex and gestational age and moderate when the head circumference z-score was ≤−2 and >−3 standard deviations. The child’s length was measured using a horizontal stadiometer. Short stature was defined as a height of <2 standard deviations below the mean for age and sex. Weight was evaluated on a platform-calibrated, precision digital scale. Because the children were unable to remain in an upright position without assistance, they were evaluated in terms of the following weight difference: Adult assistant’s weight plus child’s weight minus adult assistant’s weight. As recommended by WHO, obesity was defined based on the body mass index (BMI) calculated using weight and height and assessed relative to the WHO Child Growth Standards [19]. Overweight and obesity were respectively defined as a weight-for-height >2 and >3 standard deviations above the median for age and sex [20]. Pubertal staging was defined in accordance with the Tanner classification [21]. Testicular volume was defined by means of palpation and measured using a Prader orchidometer. Ophthalmological evaluations were performed by ophthalmologists of the Altino Ventura Foundation (Recife, Pernambuco, Brazil). The optic nerve was assessed using magnetic resonance imaging (MRI).

In addition, the study team collected blood from participants after 8 h of fasting, between 7:00 a.m. and 10:00 a.m., but not more than 2 h after the child woke up. Blood samples were tested for ultra-sensitive thyroid stimulating hormone (TSH), free-thyroxine (T4L), cortisol at 8:00 AM, insulin-like growth factor (IGF)-1, luteinizing hormone (LH), and dehydroepiandrosterone sulfate (SDHEA) using electrochemiluminescence method in a single specialized laboratory. Central hypothyroidism was defined as serum TSH concentration that is not appropriately elevated (normal range: 0.4–5.5 mIU/mL) associated with a serum-free thyroxine level of <0.77 ng/100 mL. Primary hypothyroidism was defined as a serum TSH level > 5.5 mIU/mL with a serum-free thyroxine level within or below this range (0.77–2.19 ng/100 mL). Adrenal insufficiency was defined as a baseline cortisol value below 3 µg/100 mL. Growth hormone (GH) deficiency was defined according to the measurement of IGF-1, as <27 ng/mL for males and <33.5 ng/mL for females. Stimulus tests were not done because of the risk of side effects. Early puberty and its variants were defined as laboratory evidence of LH baseline elevation ≥0.3 mIU/mL, with or without an association with increased breast volume in girls or increased testicular volume in boys. Early pubarche was defined as a puberty variant characterized by the presence of pubic hair in girls under 8 years old and in boys under 9 years old. Premature adrenarche was defined as an elevation of SDHEA levels >40 µg/dL before 8 years of age for boys and girls. Isolated premature thelarche was defined as unilateral or bilateral isolated breast development, without the development of other sexual characteristics before 8 years of age.

Statistical analyses were performed using STATA SE version 14.2 (College Station, TX, USA). Categorical variables were summarized as absolute numbers and percentages. Continuous variables were summarized as means and standard deviations.

## 3. Results

Thirty cases with Zika-related microcephaly were referred for evaluation of suspected endocrinological dysfunction at a mean age of 42 months. Notably, 29 (97%) of the cases presented with severe microcephaly. Of the 30 children, 21 had serological confirmation (20 in CSF and 1 in the serum) for Zika infection. Biological, anthropometric, and neurological characteristics of the children with Zika-related microcephaly are described in Table 1. 

Of the 30 children, 23 underwent neurological examination and presented neurological abnormalities, the most frequent being the presence of signs of pyramidal release, altered tone and inadequate visual response. Of these, 21 children underwent brain magnetic resonance that showed corpus callosum hypoplasia in 95% and optic nerve hypoplasia in 28% (Table 1). Among the 30 children evaluated, there was a report of seizures for 26 and 27 had an electroencephalogram performed. The most frequently findings of the electroencephalogram tracings of the 25 with abnormalities were focal epileptiform abnormalities, followed by multifocal epileptiform abnormalities. All were using antiepileptic drugs (AEDs) at the time of the evaluation. AEDs that these children were using included levetiracetam, clobazam, valproic acid, nitrazepam, carbamazepine, oxcarbazepine, topiramate, lamotrigine, and phenobarbital. 

Out of the 30 children, 21 underwent a brain MRI and, of these, 20 had corpus callosum hypoplasia. Of these 20 children with corpus callosum hypoplasia, 6 had optic nerve hypoplasia, 4 presented an altered pituitary image, and 2 of the later had optic nerve hypoplasia. 

Based on the medical histories, there were no reports of hypoglycemia at birth, while eight of the 30 children were reported to be jaundiced at birth. Of the children with known birth data, 13 were born SGA. Seventeen (57%) of the children with Zika-related microcephaly showed only one manifestation of endocrine dysfunction. The types of endocrine dysfunction presented alone and in combination are described in Table 2.

A detailed clinical and laboratorial characterization of the 30 children with Zika-related microcephaly is presented in the Table 3.

Early puberty variants were observed in 19 children, with similar distribution between the sexes (10 girls and 9 boys). When each variant was analyzed separately, early pubarche was observed in eight girls and two boys and high LH levels in two girls and five boys. Among these five boys, three also had hypothyroidism. Of the seven children with high baseline LH levels, none had increased testicular or breast volume, and only one had pubarche associated. Only one girl presented an increase in breast volume associated with pubarche, with Tanner M2P3 stage being observed. None of the girls had reports of genital bleeding. Variant of early puberty was observed in eight of the thirteen children who were born SGA.

All of the eight children with hypothyroidism at the time of the clinical evaluation had presented with normal neonatal screening test results for congenital hypothyroidism. One of the children with hypothyroidism had received this diagnosis previously and was being medicated with thyroid hormone.

Regarding the two children who presented with obesity alone, both were born SGA and had corpus callosum hypoplasia, and one of them had optic nerve hypoplasia and pituitary alteration in imaging examination. The other four obese children who had early puberty variants associated, three these had corpus callosum hypoplasia and optic nerve hypoplasia associated.

Among the 12 children with short stature, eight had corpus callosum hypoplasia and, of these, three had also optic nerve hypoplasia. Five of the 14 boys evaluated had cryptorchidism and one of these had already undergone bilateral orchidopexy before June 2018. 

## 4. Discussion

This is the first study on children with Zika-related microcephaly to assess the presence of endocrine dysfunction. We found endocrine dysfunction, based on clinical and laboratory alterations, in 30 children with suspected endocrinopathy who were evaluated. The most frequent endocrine dysfunctions observed were puberty disorders with early puberty variants, hypothyroidism, short stature, and obesity.

These dysfunctions occurred alone or in combination. Among the combinations, early puberty variants with hypothyroidism was the most frequent followed by early puberty variants with short stature. These findings suggest that endocrine manifestations may be part of the spectrum of CZS and may appear later in child development.

Almost all the children with endocrine dysfunction had severe microcephaly. Since the brain regulates neuroendocrine activity [22,23,24], the presence of these endocrine dysfunctions may be due to brain damage affecting the hypothalamus–pituitary axis. 

The only child with moderate microcephaly had early pubarche. We found this manifestation, of early puberty variant predominantly in females, as described in the literature [25,26,27]. However, because this was a case series of children referred for endocrinological evaluation, we cannot rule out the possibility that selection bias might have occurred.

The presence of certain neurological lesions like nystagmus and corpus callosum hypoplasia, common among the children with Zika-related microcephaly, may point towards alterations in the functioning of the hypothalamic–pituitary axis [28,29,30,31]. 

Among the twenty children with corpus callosum hypoplasia, six also had optic nerve hypoplasia, which strengthens the idea that cerebral and ocular abnormality may be associated with hypothalamus–pituitary axis dysfunction, as described in the condition of septal-optic dysplasia [10,28,29]. These patients should be carefully evaluated and monitored for this condition.

In our case series, only four of the twenty neuroimaging exams with assessment of the pituitary gland were altered with an increase in the volume of the gland, and three of these children had some manifestation of early puberty variants. However, the normal pituitary gland observed on resonance does not rule out the possibility of dysfunction of the hypothalamic–pituitary axis. Birkebaek et al. in their study of children without ZikV-related infection, reported that early puberty occurred more frequently with a structurally normal pituitary gland [32].

Early adrenarche is generally described in the literature as the most frequent cause of early pubarche [25,26]. However, in our series, only two of the ten children with pubarche presented an association with early adrenarche, suggesting that there may be another mechanism regulator of activation and/or inhibition of these puberal events. Early adrenarche and pubarche usually have benign non-progressive courses, and long-term follow-up is needed in order to clarify the behavior of these puberty disorders in these children with Zika-related microcephaly.

None of the seven children who presented high baseline LH levels had increased breast or testicular volume. Although high baseline LH levels suggest that central activation of the hypothalamus–pituitary–gonadal axis has occurred, the physical examination findings did not confirm central early puberty. However, these findings are not sufficient to definitively determine or rule out this central activation, and additional monitoring of these children is necessary. Almost half of the children in our series were born SGA. Ten of the thirteen SGA infants achieved catch-up. This proportion was similar to what has been described in the literature. It has been reported that 85–90% of SGA infants show spontaneous recovery growth, usually in the first year after [33,34]. Among these 13 children, four had early adrenarche and, of these, two reached catch up and two did not. Children who are born SGA have a particular growth pattern that reveals adrenal influence and this activity may result in early adrenarche. SGA children with catch-up develop relatively high DHEAS levels. The progressive increase in serum SDHEA concentration that is characteristic of early adrenarche may promote bone age advancement with linear growth [35,36,37]. However, children who are born SGA may continue to have short stature [33].

The short stature among the children in our series cannot be explained by the absence of catch up, nor because of the existence of an endocrine dysfunction with abnormal GH/IGF axis. Postnatal growth is also influenced by environmental factors, dietary practices and genetic potential [38,39]. In our evaluation, we did not have data on parental height.

Making a diagnosis of growth hormone deficiency (GHD) is not simple. These children have anthropometric parameters (height < two standard deviations below mean for a given age and sex) and brain malformations, and despite the lack of laboratory confirmation of GH deficiency, abnormalities in the GH/IGF axis cannot yet be surely discarded. Long term follow-up is required.

We found cryptorchidism in 35% of the boys evaluated, and 40% of them were born SGA. The exact etiology of cryptorchidism is still unknown, but among the factors involved, there is failure in the action of androgens under the control of the hypothalamus–pituitary–gonadal axis [40,41,42,43]. Additionally, as ZIKV has sexual transmission and can be detected in the testicles [44,45], this tissue tropism with viral persistence in the testicular tissue as well as direct injury to the gubernacle would be another possible hypothesis to explain the higher incidence of cryptorchidism in this group of children with ZIKV-related microcephaly [46].

Among the combined forms of endocrine dysfunction, hypothyroidism and early puberty was the most frequent combination. Although hypothyroidism usually causes delayed puberty, sporadic cases may occur with early puberty [47,48]. One child presented hypothyroidism in association with short stature, and hypothyroidism may have contributed to this child’s short stature.

Among the 30 children in our series, we found hypothyroidism in eight with high serum TSH and normal free T4 concentrations, which characterizes primary thyroid disease. The mothers of these eight children reported that the neonatal screening for congenital hypothyroidism had shown normal results. Viral infections have been cited as one of the factors involved in autoimmune thyroid diseases [49]. Congenital rubella syndrome has been correlated with autoimmune diseases such as thyroid dysfunction [12], which may manifest years after congenital infection [50]. Our findings may indicate that ZIKV is implicated as another viral agent responsible for triggering thyroid dysfunction; however, further studies are needed to confirm this hypothesis.

About 86% of the children were taking anti-epileptic drugs (AEDs) at the time of the evaluation. These drugs can have endocrine side effects. The AEDs that these children were using included levetiracetam, clobazam, valproic acid, nitrazepam, carbamazepine, oxcarbazepine, topiramate, lamotrigine, and phenobarbital. AEDs may affect thyroid function [51,52]. Phenobarbital, carbamazepine and valproate are more likely to cause such side effects. Valproate may cause weight gain and may also lead to androgenization with increased serum testosterone concentrations and menstrual disorders [53,54,55].

The results of our study need to be interpreted with caution. As already stated, the cases of hypothyroidism we observed were characterized as primary thyroid disease. The findings of short stature did not appear to be due to GH deficiency, although we were unable to rule out the possibility that this may have been due to GH/IGF axis dysfunction. Also, based on the assessment made so far, it was not possible to affirm or rule out the possibility that puberal events might have been associated with a central cause. Despite the presence of severe microcephaly with midline brain malformations, with or without associated optic nerve abnormalities, it was not possible to attribute the endocrine dysfunctions that were found to hypothalamus–pituitary axis involvement. Half of the children in this sample were born SGA, which is a component of CZS, and the vast majority were using anticonvulsant drugs, which are risk factors for some of these disorders. Nor could we rule out the notion that direct action by ZIKV might have affected the tissues responsible for the functioning of the endocrine system. Thus, we cannot yet say that these endocrine dysfunctions express central activation of the hypothalamus–pituitary axis.

This series of 30 children showed manifestations for which there were no previous reports. This study is of public health relevance because it points to the pediatricians the need to systematically investigate endocrine disorders in children with Zika-related microcephaly. Selection bias may have been a possible limitation of this study, since the children were referred for endocrine evaluation due to endocrinological manifestations. The clinical evaluation performed by the pediatrician who referred the children to the endocrinologist may have missed mild symptoms. As hormonal measurements were not performed in all children with Zika-related microcephaly of MERG-PC, hormonal alterations may have not been detected. If children did not present evident endocrine manifestations, this may have led to underdiagnosis of cases and consequently underestimated the number of children with endocrine dysfunction and failed to apprehend the spectrum of these disorders. However, it should be noted that this is a relatively high number of children with endocrine disorders if we take into account that most of them were originated from the MERG-PC which follows approximately 200 children with Zika-related microcephaly. We did not include sampling and testing healthy control children. Therefore, hormonal levels were not compared those of a healthy control group and we used the established standards for hormonal level assessment. Laboratory evaluation was another limitation, because only baseline hormone assaying was performed. Levels of hormones were not measured in mothers during pregnancy and breast feeding; maternal hormonal levels can affect levels in fetuses and subsequently in children. However, it is not likely that maternal endocrine dysfunction would affect these children in this proportion and diversity. In addition, provocative tests were not performed. Stimulus tests to assess the growth axis in children with brain malformations can result in seizures and lack safety and accuracy. Other tests that have been deemed necessary for better evaluation of endocrine dysfunctions will be performed in the follow-up of these children. As the hormonal evaluation was performed only at a one-time point, longitudinal study with more frequent sampling and the addition of other tests will provide a better dynamic understanding of hormonal dysfunction.

## 5. Conclusions

This case series is the first study in the literature to discuss endocrine disorders on children with Zika-related microcephaly, providing the first evidence and clues that these disorders may be a component of the spectrum of congenital Zika syndrome, that may be further explored in longitudinal analytical studies with larger sample sizes. The evaluation of the presence of subclinical or unsuspected endocrine disorders, the incorporation of new hormone measurements and the comparison with the appropriate control group will complement our findings. Careful monitoring of children with Zika-related microcephaly will allow early diagnosis and intervention.

## Figures and Tables

**Table 1:** Neurological characteristics, age, sex distribution and anthropometric characteristics.

Parameters	Values	
Age, sex distribution and anthropometric characteristics		
Age at diagnosis (Months)	41.66	(±1.29)
z-score current head circumference	−5.89	(±1.84)
Sex [female (%)/male (%)]	16/14	[(53.33)/(46.67)]
Weight (kg)	14.608	(±3.941)
Height (cm)	92.6	(±4.40)
Height standard deviation score	−1.70	(±1.26)
Body mass index (kg/m^2^)	16.79	(±3.98)
Body mass index standard deviation score	1.07	(±2.63)
z-score for head circumference at birth	−3.95	(±1.93)
Neurological characteristics		
Seizure	26/30	86.6%
Electroencephalogram without abnormalities	2/27	7%
Electroencephalogram abnormalities:		
Focal epileptiform	10/27	37%
Multifocal epileptiform	7/27	26%
Generalized epileptiform	5/27	19%
Multifocal and generalized epileptiform	2/27	7%
Focal and generalized epileptiform	1/27	4%
Neurological abnormalities	23/23	100%
Signs of pyramidal release	23/23	100%
Altered tone	22/23	96%
Localized motor deficit	1/23	4%
Inadequate visual response	18/23	30%
Inadequate auditory response	7/23	30%
Brain Magnetic Resonance Imaging	21/30	70%
Hypoplasia of corpus callosum	20/21	95%
Altered pituitary image	4/20	20%
Hypoplasia of optic nerve	6/21	28.6%
Nystagmus	19/30	63.3%

Data are given as mean ± standard deviation or median (25th–75th percentile).

**Table 2:** Endocrine dysfunctions seen alone and in combination among children with ZikV-associated microcephaly in in the Microcephaly Epidemic Research Group (MERG) Cohort evaluated between February and June 2019 in Recife, Pernambuco, Brazil (N = 30).

Endocrine Dysfunction	N	%
Early puberty, pubarche, adrenarche	8	26.67%
Short stature	5	16.67%
Hypothyroidism	2	6.67%
Obesity	2	6.67%
Early puberty + hypothyroidism	4	13.33%
Early puberty + short stature	3	10%
Short stature + hypothyroidism	1	3.33%
Short stature + adrenal insufficiency Early puberty + obesity + short stature	1 2	3.33% 6.67%
Early puberty + obesity + adrenal insufficiency Early puberty + obesity + hypothyroidism	1 1	3.33% 3.33%
Total	30	100%

**Table 3:** Clinical and laboratory characterization of the 30 children with Zika-related microcephaly assessed with endocrine dysfunction.

Case	Sex	HC at Birth	HC	BMI	Height SDS	Tanner	LH	SDHEA	TSH	T4 Free	IGF−1	Glucose	Cortisol
Early puberty variants													
1	F	−4.96	−6.28	1.24	−0.89	M1P3	0.1	18	2.62	1.39	295	87	17.2
2	M	NI	−6.19	0.22	−1.61	G1P1	0.1	49	4.08	1.47	186.8	89	8.3
3	M	−4.69	−6.14	1.86	−0.34	G1P2	0.1	72	1.4	1.34	247.7	95	13
4	F	−2.43	−5.24	0.57	−0.6	M1P2	0.1	29	4.24	1.01	218.8	80	5.4
5	F	−2.85	−2.61	−1.1	1.97	M1P2	0.58	3	2.23	1.52	172.1	84	4.8
6	F	−2.01	−4.96	0.05	−0.58	M1P2	0.1	7	4.36	1.21	226.9	90	13.6
7	M	−4.3	−5.1	1.79	−0.83	G1P2	0.1	9	3.68	0.92	178.8	95	7.6
8	M	−6.88	−8,89	−2.72	−1.46	G1P1	0.1	59	1.99	1.42	147.3	99	9.7
Short stature													
9	F	−3.27	−6.31	−2.87	−2.36	M1P1	0.1	30	3.24	1.2	146.1	85	20.4
10	M	−5.94	−4.11	−1.77	−3.79	G1P1	0.1	12	1.81	1.23	160.4	74	8.1
11	M	NI	−9.21	0.98	−2.95	G1P1	0.1	13	1.99	1.25	187.7	89	6.4
12	F	−4.96	−6.26	−1.18	−2.81	M1P1	0.1	18	5.07	1.85	196.2	85	6.2
13	F	−4.12	−6.26	0.11	−3.44	M1P1	0.1	2	1.84	1.28	230.1	87	7.9
Hypothyroidism													
14	F	−1.59	−4.89	−1.24	0.02	M1P1	0.1	1	7.69	1.43	165.2	86	7.1
15	F	−3.07	−3.41	−0.83	−0.84	M1P1	0.1	6	9.55	1.5	85.4	81	13
Obesity													
16	F	−4.54	−5.66	3.48	−1.78	M1P1	0.1	6	6.26	1.18	261.1	89	9.6
17	M	−5.09	−3.96	4.84	−1.2	G1P1	0.1	11	1.05	1.44	75.2	111	5.6
18	M	−4.3	−5.09	7.04	−1.55	G1P1	0.1	12	2.23	1.2	216.7	81	24
Hypothyroidism and Variants of Early Puberty													
19	F	−4.12	−4.91	2.36	−0.9	M1P2	0.1	8	9.86	1.28	71.7	73	4.1
20	M	−3.12	−4.14	2.47	−0.7	G1P1	0.36	3	4.69	1.35	95.5	83	6.4
21	M	−3.51	−6.48	0.22	−1.57	G1P1	1.22	25	13.6	1.42	91	80	9.8
22	M	NI	−4.1	2.33	−1.51	G1P1	0.58	17	6.14	1.24	153.5	82	12.9
Short stature and Variants of Early Puberty													
23	M	−1.62	−5.48	−2.07	−2.77	G1P1	0.3	14	4.45	1.43	254.2	91	5
24	F	−4.96	−7.33	−0.05	−3.19	M1P2	0.1	11	3.3	1.41	269.4	94	
25	F	−4.12	−7.32	1.03	−2.21	M1P2	0.1	76	1.48	1.38	229.3	88	4.7
Short stature and adrenal insufficiency													
26	F	−1.59	−3.77	0.28	−2.89	M1P1	0.1	0	3.59	1	194.9	81	1.5
Short stature and Hypothyroidism													
27	M	NI	−8.22	−3.45	−2.84	G1P1	0.1	18	5.81	1.21	195	86	7.8
Obesity, Variants of Early Puberty and adrenal insufficiency													
28	F	−6.65	−10.5	7.78	−1.46	M1P2	0.1	3	4.48	1.3	199.1	85	0.4
Obesity and Variants of Early Puberty													
29	M	−3.91	−5.42	3.03	−2.37	G1P1	0.35	1	3.84	0.88	175.1	94	6.4
30	F	NI	−8.52	3.63	−3.72	M1P1	0.1	45	2.08	1.22	195	81	8.6

HC: Head Circumference; BMI: Body mass index, SDS: Standard Deviation Score; LH: Luteinizing Hormone; SDHEA: Dehydroepiandrosterone Sulfate; TSH: Thyroid-stimulating Hormone; T4L: Free thyroxine; IGF-1: Insulin-like Growth Factor-1; NI: Not Informed.
